# Alterations of the 70 kDa heat shock protein (HSP70) and sequestosome-1 (p62) in women with breast cancer

**DOI:** 10.1038/s41598-021-01683-8

**Published:** 2021-11-15

**Authors:** Theofano Orfanelli, Spyridon Giannopoulos, Eleni Zografos, Aikaterini Athanasiou, Ann Marie Bongiovanni, Georgios Doulaveris, Tracy-Ann Moo, Dayle LaPolla, Chris N. Bakoyiannis, Georgios E. Theodoropoulos, Georgios C. Zografos, Eleni Andreopoulou, Steven S. Witkin

**Affiliations:** 1 First Department of Propaedeutic Surgery, Hippocration General Hospital, National and Kapodistrian University of Athens, Athens, Greece; 2 First Department of Surgery, Laikon General Hospital, National and Kapodistrian University of Athens, Athens, Greece; 3 Department of Clinical Therapeutics, Alexandra Hospital, National and Kapodistrian University of Athens, Athens, Greece; 4 Department of Obstetrics & Gynecology, Weill Cornell Medicine, New York, NY USA; 5 Department of Surgery, Weill Cornell Medicine, New York, NY USA; 6 Department of Medicine, Weill Cornell Medicine, New York, NY USA; 7 First Department of Surgery, Division of Vascular Surgery, Laikon General Hospital, National Kapodistrian University of Athens, Athens, Greece

**Keywords:** Cancer, Immunology

## Abstract

Peripheral blood mononuclear cells (PBMCs) respond to altered physiological conditions to alleviate the threat. Production of the 70 kDa heat shock protein (HSP70) is up-regulated to protect proteins from degradation. Sequestosome-1 (p62) binds to altered proteins and the p62-protein complex is degraded by autophagy. P62 is also a regulator of intracellular kinase activity and cell differentiation. We hypothesized that the PBMC response to a malignant breast mass involves elevated production of HSP70 and a decrease in intracellular p62. In this study 46 women had their breast mass excised. PBMCs were isolated and intracellular levels of HSP70 and p62 were quantitated by ELISA. Differences between women with a benign or malignant breast mass were determined. A breast malignancy was diagnosed in 38 women (82.6%) while 8 had a benign lesion. Mean intracellular HSP70 levels were 79.3 ng/ml in PBMCs from women with a malignant lesion as opposed to 44.2 ng/ml in controls (p = 0.04). The mean PBMC p62 level was 2.3 ng/ml in women with a benign breast lesion as opposed to 0.6 ng/ml in those with breast cancer (p < 0.001). Mean p62 levels were lowest in women with invasive carcinoma and a positive lymph node biopsy when compared to those with in-situ carcinoma or absence of lymphadenopathy, respectively. Intracellular HSP70 and p62 levels in PBMCs differ between women with a malignant or benign breast lesion. These measurements may be of value in the preoperative triage of women with a breast mass.

## Introduction

The malignant transformation of cells anywhere in the body is almost always accompanied by changes in systemic immunity. Alterations in antigen expression and perturbations in the local environment are detected by the immune system and result in changes in the properties of immune cells^1^. The ability to identify immune cell alterations that predict malignancy will be of value in the initial screening of individuals with a suspected malignancy.

Autophagy is an intracellular mechanism present in almost all organisms to maintains cell functions and optimizes survival. Aggregated or non-functional proteins and organelles become bound to a cytoplasmic protein, sequestosome-1 (p62)^2,3^ and the complex is sequestered in a double-membraned structure called an autophagosome^4,5^. The autophagosome merges with a lysosome and the engulfed components along with p62 are degraded by lysosomal enzymes. The amino acid, nucleoside, carbohydrate and lipid components are returned to the cytoplasm for reutilization^6–11^. The variation in p62 concentration in the cytoplasm has been used as a biomarker for the extent of autophagy induction^12–14^. Intracellular p62 also modulates the functions of various kinases and participates in cell activation and differentiation^15–17^. Therefore, the various p62-related activities must all be taken into consideration when evaluating the consequences of alterations in its intracellular concentration.

An additional mechanism that promotes cell survival under different conditions is activation of the gene Heat Shock Protein Family A Member 1A (HSPA1A), coding for the 70 kDa heat shock protein (HSP70). When a cell encounters non-physiological conditions, HSP70 synthesis is greatly up-regulated. The HSP70 binds to nascent peptides and promotes the formation of functional proteins. It also prevents protein denaturation^18–20^. The HSP70 is also released from the stressed cell and functions in the extracellular milieu to activate pro-inflammatory immunity to combat the perceived stress^21^. Numerous studies have reported on elevations in circulating HSP70 in association with malignant transformation, including breast cancer^22–25^.

While multiple studies have evaluated changes in autophagy and HSPA1A expression in mammary cells that are associated with malignancy^26–29^, concomitant potential alterations in HSP70 and p62 in peripheral blood mononuclear cells (PBMCs) have not been evaluated. In this exploratory study we compared the level of p62 and HSP70 in PBMCs from women with malignant and benign breast lesions. We hypothesized that the PBMC response to a malignant breast mass involves alteration of HSP70 and p62, which may serve as biomarkers for the prognosis of breast cancer patients.

## Material and methods

### Patient enrollment

Women with a breast mass referred to the Section of Breast Surgery at Weill Cornell Medical College who underwent diagnostic breast surgery from 2015 to 2017 and consented to have their PBMCs obtained and evaluated, were enrolled in the study. All patients had preoperative confirmation of a breast mass by sonography, mammogram or magnetic resonance imaging followed by a fine needle aspiration biopsy or a core biopsy. Women with a cancer diagnosis underwent surgery for surgical staging. Women with a benign breast biopsy underwent surgery either for definitive tissue diagnosis, especially for large lesions, or for management of symptoms. All women with a previous history of any type of malignancy or neoadjuvant chemotherapy, with an active infection or a history of a chronic inflammatory disease or who did not undergo surgical evaluation of their mass were excluded. This study was approved by the Institutional Review Board of Weill Cornell Medical College and was conducted according to Declaration of Helsinki. All subjects provided written informed consent at their initial preoperative evaluation.

The following baseline clinicopathologic characteristics were collected from each subject: age, histology, histology subtypes, Estrogen Receptor (ER) status, Progesterone (PR) status, Human Epidermal growth factor Receptor 2 (HER2) status, grade, sentinel lymph node status, and stage. In all cases, the diagnosis of breast cancer was made by a clinical pathologist based on histological tissue analysis from surgical excision of the breast mass. The personnel of the laboratory were blinded to all clinical and histological data until the completion of all experimental assays.

### PBMC isolation and intracellular hsp70 and p62 quantification

Blood was collected from a peripheral vein preoperatively into a sterile heparin-containing tube and sent to the laboratory for analysis. The isolation of PBMCs and production of cell lysates was as previously described^30^. Briefly, PBMC was isolated by Ficoll-Hypaque gradient centrifugation (GE Healthcare Biosciences, Piscataway, New Jersey). The PBMC fraction was gently removed and resuspended in Roswell Park Memorial Institute (RPMI) 1640 medium (Invitrogen, Carlsbad, California) for two cycles of centrifugation and resuspension. The final suspension was adjusted to a concentration of 5 × 10^6^ cells/ml. The PBMCs were centrifuged again and 130 μl of a cell lysate buffer (1% Triton X 100, 1% sodium deoxycholate, 0.1% sodium dodecyl sulfate, 20 mg/ml deoxyribonuclease, 100 mmol/l protease inhibitor cocktail (Sigma, St Louis, Missouri) in 50 mmol/l Tris–HCl, pH 7.4, 150 mmol/l NaCl, 1 mmol/l EDTA, and 1 mmol/l ethylene glycol tetraacetic acid) was added to the pellets. The final incubation mixture contained the same number of cells in each sample. The mixture was incubated for 30 min at 4 °C and the lysed PBMCs were centrifuged at 11,000 rpm for 10 min. The supernatant was collected and concentrations of HSP70 and p62 were quantitated using commercial ELISA kits (HSP70 ELISA kit from R&D Systems, Minneapolis, MN, p62 ELISA kit from Enzo Life Sciences, Farmingdale, New York). The lower limit of detection was 156 pg/ml for HSP70 and 100 pg/ml for p62. Values were converted to ng/ml by comparison to a standard curve generated in parallel to each assay.

### Statistical analysis

The Mann–Whitney *U* test was utilized as appropriate to compare continuous variables as the values were not normally distributed. The Pearson chi-square test or Fisher exact test was performed to assess for association between categorical variables. The software GRAPH PAD INSTAT Version 3.0 was utilized for all statistical analyses, with p-values < 0.05 considered as statistically significant.

## Results

A total of 38 patients (82.6%) women were diagnosed with a breast malignancy, and 8 (17.4%) had a benign breast lesion. Patient characteristics are summarized in Table 1. There were no differences in age, ethnicity/race, body mass index or reproductive history between the two groups. Table 2 summarizes the histopathologic characteristics of the 38 breast cancer patients. The majority were diagnosed with early stage (IA) ductal carcinoma with positive ER/PR. In 34 women (89.5%) their breast carcinoma was invasive while 4 women (10.5%) had carcinoma-in situ.

**Table 1:** Characteristics of the study population.

Characteristic	Benign (n = 8)	Malignant (n = 38)	p value
**Age, median (range)**	49.5 (34.71)	54 (31.71)	NS
**Race/ethinicity—n (%)**			NS
Non-Hispanic White	3 (37.5)	15 (39.5)	
Non-Hispanic Black	2 (25.0)	9 (18.8)	
Asian	1 (12.5)	4 (10.5)	
Hispanic	2 (25.0)	7 (18.4)	
Other	0 (0.0)	3 (7.9)	
**Body mass index (BMI)—mean (SD)**	30.2 (6.5)	30.9 (8.3)	NS
**Gravidity, median (range)**	3 (0.6)	3 (0.10)	NS
**Parity, median (range)**	2 (0.5)	2 (0.8)	NS

*NS* not significant.

**Table 2:** Histopathologic characteristics of patients diagnosed with breast malignancy.

Characteristic	No. women (n = 38)
**Histologic subtype—n (%)**	
Ductal	25 (65.8)
Lobular	9 (23.7)
Mixed (ductal and lobular)	4 (10.5)
**ER status—n (%)**	
Positive	33 (86.8)
Negative	5 (13.2)
**PR status—n (%)**	
Positive	32 (84.2)
Negative	6 (15.8)
**HER2 status—n (%)**	
Positive	6 (15.8)
Negative	29 (76.3)
Unknown	3 (7.9)
**Histologic grade—n (%)**	
1	7 (18.4)
2	16 (42.1)
3	14 (36.8)
**Sentinel lymph node—n (%)**	
Positive	8 (21.1)
Negative	28 (73.7)
Not performed	2 (5.2)
**Prognostic stage—n (%)**	
0 (in situ)	4 (10.5)
IA	20 (52.6)
IB	0 (0.0)
IIA	6 (15.8)
IIB	7 (18.4)
IIIA	0 (0.0)
IIIB	0 (0.0)
IIIC	1 (2.6)
IV	0 (0.0)

*ER* Estrogen receptor, *PR* Progesterone receptor, *HER2* Human epidermal growth factor receptor 2.

The mean intracellular PBMC HSP70 and p62 levels in all subjects is shown in Table 3. The mean HSP70 concentration was 79.3 ng/ml in women with breast cancer versus 44.2 ng/ml in the benign group (p = 0.04). Conversely, the mean intracellular p62 level was 0.6 ng/ml in women with breast cancer and 2.3 ng/ml in the controls (p < 0.001).

**Table 3:** Comparison of intracellular HSP70 and p62 in PBMCs from women with malignant or benign breast disease.

	Malignant (n = 38)	Benign (n = 8)	p value
Intracellular HSP70, (ng/ml)	79.3 ± 7.4	44.2 ± 8.6	0.04
Intracellular p62, (ng/ml)	0.6 ± 0.1	2.3 ± 0.5	< 0.01

Data are presented as mean ± SEM.

A subgroup analysis in the women with breast cancer revealed that the mean PBMC p62 level in those with invasive carcinoma (0.30 ng/ml) was significantly lower compared to those with in-situ carcinoma (1.10 ng/ml) (p = 0.01). There was no difference in the levels of intracellular HSP70 between these two groups. A sentinel lymph node biopsy was performed in 36 (94.7%) of the cancer patients and 8 (22.2%) were found to be positive. Women with a positive sentinel lymph node had a significantly lower intracellular p62 level in their PBMCs (0.04 ng/ml) as compared to those with negative lymph nodes (1.4 ng/ml) (p = 0.008). There were no differences in the concentrations of intracellular HSP70 or p62 between women with different histological types or stages of breast cancer.

## Discussion

The results of the present study demonstrate that HSP70 is elevated and the p62 level is decreased in PBMCs from women with breast cancer as compared to their levels in PBMCs from women with benign breast lesions. We also found that the p62 level was further decreased in PBMCs from patients with invasive carcinoma as compared to those with in situ carcinoma. This paralleled our observation that p62 was lower in PBMCs from breast cancer patients with a positive sentinel lymph node biopsy as compared to those with a negative sentinel lymph node biopsy.

The influence of HSP70 levels on autophagy has been investigated previously. Doklandy et al.^31^ in an in vitro study clearly demonstrated that the extent of HSPA1A expression was inversely related to the level of autophagy in tumor cell lines. The mechanism involved activation of Akt kinase and the mammalian target of rapamycin (mTOR), a kinase that blocks autophagy induction. The authors proposed that an inverse correlation might exist between HSPA1A expression and autophagy in tumor cells. Additionally, Kanninen et al.^30^ in an in vitro study provided data that the intracellular HSP70 concentration in PBMCs from pregnant women and the p62 level were inversely proportional. Other studies in yeast cells demonstrated that the chaperone activity of HSP70 might be the main mechanism to prevent accumulation of misfolded proteins in the endoplasmic reticulum and, thus, limit autophagy^32,33^.

The mechanism(s) responsible for decreased p62 levels and elevated HSP70 production in PBMCs in women with a malignant mammary tumor remains to be definitively determined. However, it is likely that this is a consequence of the appearance in the circulation of tumor-related antigens as well as intracellular macromolecules that are released from lysed cells following malignant transformation of mammary cells. These alterations in the extracellular milieu would invariably signal the need for immune system activation. The up-regulation of both a stress response and autophagy activity in PBMCs under these conditions would increase HSP70 levels and, alternatively, decrease p62 as it is consumed during autophagy. This decline in intracellular p62 might also trigger kinase-related activities that further facilitate tumor-related PBMC activation. This is consistent with our observation of a further decrease in p62 and an increase in HSP70 in PBMCs as breast cancer became more invasive. Therefore, we hypothesize that elevated intracellular levels of HSP70 and decreased p62 levels in PBMCs could be markers of breast cancer and for advanced disease**.**

A strength of our study is its unique focus on PBMCs rather than on tumor cells, and specifically on alterations in intracellular levels of compounds associated with elevated immune activation. However, the findings must be interpreted in the context of several limitations. First, the sample size of the study population, especially the group with benign breast lesions, was very small and, therefore, subject to selection bias. The majority of women with benign lesions do not typically undergo surgery which limited our ability to recruit more subjects in this category. We felt it was necessary to only include women with benign disease who underwent a surgical evaluation in order to verify their diagnosis. In addition, our study was not designed to detect differences in HSP70 and p62 levels based on histology and stage of breast cancer. Therefore, our study was under- powered to evaluate these differences and the findings must be regarded as preliminary and hypothesis-generating. Further studies are warranted to compare the preoperative and postoperative levels of these markers, since HSP70 and p62 levels may also fluctuate according to physical conditions and stresses other than breast malignancy. Lastly, as mentioned above, p62 has multiple intracellular activities and further studies are required to determine the relative alterations in each of these functions in PBMCs in women with breast cancer^30,34^.

## Conclusion

Intracellular properties of PBMCs are altered in women with a malignant breast tumor. Detection of elevated levels of HSP70 and decreased concentrations of p62 in PBMCs may have value as a diagnostic indicator of a breast malignancy or its recurrence. Additional investigations of the observations reported here might be of value in development of clinically useful tests for early detection of invasive breast cancer and/or its differentiation from benign breast masses.
